# A Practical Synthesis of Betulonic Acid Using Selective Oxidation of Betulin on Aluminium Solid Support

**DOI:** 10.3390/molecules171011849

**Published:** 2012-10-09

**Authors:** Nina Melnikova, Irina Burlova, Tatiana Kiseleva, Irina Klabukova, Marina Gulenova, Аleksey Kislitsin, Viktor Vasin, Boris Tanaseichuk

**Affiliations:** 1 Department of Pharmaceutical Chemistry, Nizhny Novgorod State Medical Academy, Minin sq. 10/1, Nizhny Novgorod 603-000, Russia; 2 Department of Chemistry, N.P. Ogarev Mordovian State University, Mironov St. 3-274, Saransk 430-005, Russia; 3 Department of Chemistry, Nizhny Novgorod State University, Kuibishev St. 17-25, Nizhny Novgorod 603-950, Russia

**Keywords:** oxidation on alumina, betulin, betulonic acid, protection of double bond by Al^3+^-ions

## Abstract

The room temperature oxidation of betulin by Cr(VI) compounds in aqueous acetone on solid supports such as alumina, zeolites and silica gel has been studied. The oxidation on alumina support leaded to a single product—betulonic acid—in quantitative yield. One hundred percent selective oxidation during 30 min of betulin up to betulonic aldehyde was determined when silica gel support was used. The oxidation of betulin using zeolites as a support gives a mixture of betulonic acid and aldehyde in a 2:1 ratio. It is proposed the selective oxidation up to betulonic acid is due to the influence of Al^3+^-ions.

## 1. Introduction

Betulonic acid [lup-20(29)-en-3-oxo-28-oic] has valuable biological properties such as antiviral, antitumor, anti-inflammatory, antimicrobial, hepatoprotective, as well as immunostimulant activities [1,2,3,4,5]. 

Until the 2000s interest to betulonic acid was primarily due to its role as the precursor for synthesis of betulinic acid, which is an effective drug against human melanoma. As a rule, betulonic acid was produced using betulin oxidation by Jones’ reagent (CrO_3_/H_2_SO_4_/acetone) or similar synthesis routes with yields less more 75%. This synthesis is long, not very profitable and requires challenging purification steps, including column chromatography, multiple recrystallizations and extraction using very large volumes of solvents, making it unsuitable for extensive scale industrial application [1,2,6,7,8,9,10].

**Figure 1:**
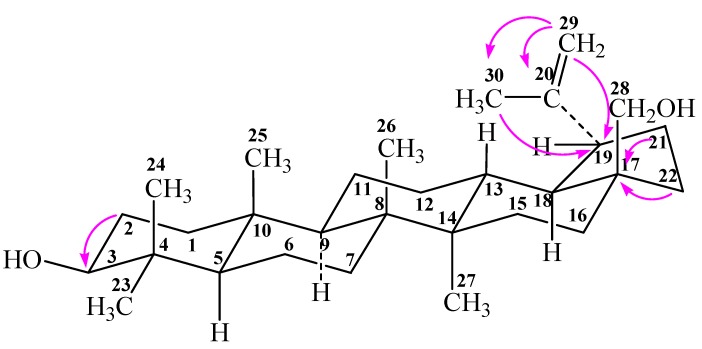
Structure of betulin (**1**).

The main problems in the oxidation of betulin are due to, firstly, nonselectivity of the process, because the molecule contains three active centers: primary (at C-28) and secondary (at C-3) alcohol groups and an isopropenyl moiety. Secondly, it is difficult to regulate the range of oxidation because products may be aldehydes or ketones as well as acids. Thirdly, the lability of the betulin structure (Figure 1) provides conditions for different rearrangements as follows on the basis NOESY spectra calculation taking into account of Overhauser effect [11,12]. 

Taking into account the features of the betulin structure, historically oxidation selectivity was achieved in synthetic routes with protection of the primary alcohol groups of betulin, for example, by acetylation using acetic anhydride [13]. Then betulin diacetate was hydrolyzed up to betulin monoacetate at the C-3 position. The non-protected C-28 alcohol group is oxidized to a carboxyl group by CrO_3_ in acetic acid and betulinic acid is formed after removing the acetate protective groups. 

A number of new chromium (VI)-containing compounds with heterocyclic bases, like pyridinium chlorochromate, pyridinium bromochromate, quinolinium chlorochromate, quinolinium fluoro-chromate, quinolinium bromochromate, imidazolium fluorochromate, pyridinium fluorochromate, imidazolium dichromate and quinolinium dichromate have been developed to improve the selectivity of oxidation of organic compounds [14,15,16].

Quite recently, a two-step route [17] utilizing solid-supported chromium oxide and potassium permanganate has been suggested and even more recently, a TEMPO-mediated electrochemical approach has been devised [7]. Whereas the oxidation of betulin with TEMPO (2,2,6,6-tetramethyl-piperidin-1-oxyl)/NaClO_2_/NaOCl at 35 °C furnished 92% of the betulinic aldehyde (**4**), the reaction of betulin with 4-acetamido-TEMPO/NaClO_2_/NaOCl at 50 °C gave betulinic acid in an 86% isolated yield. Both of these approaches, however, are small-scale preparations and the obtained yields only moderate. The increased selectivity of betulin oxidation was developed only for betulinic acid or betulinic aldehyde production. 

Betulin oxidation to betulonic acid must be similar to the oxidation of steroid hydroxyls. Usually the oxidation of steroid or other natural triterpenes having hydroxyl groups at the C-3 position by chromic acid proceeds selectively in the presence of acetone, but the reaction gives double bond oxidation products if acetone is changed for other solvents. The oxidation of primary alcohol groups by chromic acid in acetone medium (Oppenauer’s oxidation) may proceed up to aldehydes or acids like by isopropyl Al-salt catalysis [18]. Concurrent directions of oxidation characteristics didn’t seem reasonable for practical synthesis of betulin derivatives, consequently, development of a practical synthesis of betulonic acid with a mild, selective and high-yielding oxidation in the first stage is a very important problem. Accordingly, several methods have been recently reported for the environmentally benign oxidation of primary and secondary alcohols to carbonyl compounds using solid supports [17,19,20].

Recently the possibility of high selective oxidation of betulin by chromium (VI) compounds immobilized on aluminium oxide or silica gel solid support in donor solvents up to betulonic acid as a precursor of betulinic acid was shown [21]. In this paper we study the protective function of surface aluminum ions as the reason behind the high yields seen in the practical synthesis of betulonic acid from betulin at room temperature in a one-pot reaction by using low cost, easily available potassium dichromate (K_2_Cr_2_O_7_) which may be regenerated as oxidant in the presence of H_2_SO_4_ and acetone on a solid support such as alumina. 

## 2. Results and Discussion

It is well known that Lewis acids may catalyze the oxidation of alcohols up to aldehydes [22]. The most catalytic activity was shown by aluminum salts, being also the most selective catalysts. In the first stage of our study the effect of Al^3+^ on oxidation selectivity and conversion of betulin (**1**) was demonstrated in experiments by using Al_2_(SO_4_)_3_ in aqueous acetone at room temperature. We obtained betulonic acid (**2)** as a single product in quantitative yield at a molar ratio ν_Cr__6+_/ ν_betulin_ equal to 3, and it was easily separated out after 1.5–3.0 h by adding water to the supernatant liquid. The crude betulonic acid didn’t require challenging purification, if it was used for synthesizing betulonic acid derivatives, for example, betulinic acid. The synthesis of betulinic acid by NaBH_4_ reduction of betulonic acid without challenging purification in isopropanol or THF medium is described in the Experimental section.

In the case when Al_2_(SO_4_)_3_ was absent in the reaction mixture, conversion of betulin was only 40% with the formation of two main products—betulonic acid **2** and betulonic aldehyde **3**—and several undetermined products (Table 1). It may be supposed that they were the results of isopropenyl moiety oxidation as estimated by ^13^C-NMR-spectra [decrease of the signals with δ = 109 ppm (C-29) and δ = 150 ppm (C-20)]; and by IR-spectroscopy [decreasing intensity of bands at 882 cm^−1^ (methylene terminal double bond) and 1642 cm^−1^ (double bond)].

**Table 1:** Time distribution of selectivity in betulin oxidation without Al_2_(SO_4_)_3_ at molar ratio ν _Cr_^6+^/ ν_betulin_ equal to 3 in acetone at room temperature.

Time, min	Conversion of betulin (1), %	Products in reaction mixture, % (HPLC, IR and NMR-spectra control)			
		Betulinic aldehyde (4)	Betulonic aldehyde (3)	Betulonic acid (2)	Other unknown products
2	5	15–20	55	–	20–25
5	7	15	50–60	–	20–25
10	12	5	60	10	25
20	17	–	60	10	30
65	25	–	50	20	30
80	30	–	40	30	30
160	37	–	35	35	30
300	40	–	20	50	30
400	50	–	–	70	30
150 *	100	–	0–5	95–100	–

*: The experiment with Al_2_(SO_4_)_3_.

The feature of chromium oxidation in the presence of Al^3+^, in addition to high selectivity, was the acceleration of the reaction: 100% betulin conversion was achieved in 150 min. This may mean aprotective role of Al^3+^ as well as a catalytic effect. The disadvantage of this high selective oxidation in the presence of Al_2_(SO_4_)_3_ was the formation of a green coloured inorganic precipitate of Cr_2_(SO_4_)_3_ with toxic properties. 

It is a very interesting approach to use a solid support containing Al^3+^-ions due to the high oxidation selectivity by using Cr^3+^ sorption on a solid support to minimize sediments. The experiments with aluminosilicate (zeolites) with similar dispersity as a granular solid support were carried out to demonstrate the role of Al_2_O_3_ as the best regulator of selective betulin oxidation. SiO_2_ was chosen as solid support with other acid properties than the Lewis acid—Al^3+^.

Selectivity of oxidation and conversion of betulin (**1**) on alumina support in aqueous acetone was due to molar ratio ν_Cr6+_/ν_betulin_, mass ratio m_solid support_/m_betulin_, duration of processes and didn’t depend on the Cr^6+^-containing compounds (Table 2, examples 3–5). The best selectivity of betulin oxidation up to betulonic acid (**2**) (100%) with high yield (93%–98%) was obtained under the conditions of experiments 3a–c. The decrease of selectivity in betulin oxidation was shown in experiments with zeolites (Table 2, example 6), since the surface concentration of Al^3+^ in zeolite is much less than in alumina. 

**Table 2:** Oxidation of betulin (0.02 mol·L^−1^) with Cr^6+^-containing compounds on solid support in aqueous acetone at 15–25 ^ο^C during 1.5–3 h.

Example	Oxidant				Conversion of betulin 1, %	Ratio 2:3	Selectivity, %
	Cr6+	Solid support	ν Cr6+	msolid support/ mbetulin			
			νbetulin				
1	K_2_Cr_2_O_7_	Al_2_(SO_4_)_3 _*	3:1	–	100	100:1	100 (2)
2	K_2_Cr_2_O_7_	–	3:1	–	40	1:1	50 **
3а	K_2_Cr_2_O_7_	Al_2_O_3_	3:1	6:1	100	100:1	100 (2)
3b	K_2_CrO_4_						
3c	CrO_3_						
4	K_2_Cr_2_O_7_	Al_2_O_3_	1.5:1	6:1	100	3:1	75 (2)
5	K_2_Cr_2_O_7_	Al_2_O_3_	1.5:1	3:1	80	1:1	50
6	K_2_Cr_2_O_7_	zeolites	3:1	2:1	100	7:3	70 (2)
7 ***	K_2_Cr_2_O_7_	SiO_2_	3:1	10:1	100	1:100	100 (3)

* C_Al2(SO4)3_ = 1.6 mmol·L^−1^; ** selectivity was estimated and the oxidation of isopropenyl moiety wasn’t taken into account; *** synthesis during 30 min.

This result was different in comparison with the experiments carried out without aluminum-ions (Table 2, example 2) or Al_2_O_3_-produced ones but under other conditions being equal (Table 2, examples 3–5). The reaction time increased up to 3–5 h (80%–100% betulin conversion), if the molar ratio ν _Cr_^6+^/ν_betulin_ was decreased down to 1.5:1 (Table 2, examples 4–5). At the same time the selectivity of betulin oxidation was decreasing (Table 2). The colourless flakes of inorganic nature were formed immediately, and they became green. The flakes adhered to the alumina granules increasing in size, and the supernatant liquid became more homogeneous.

**Figure 2:**
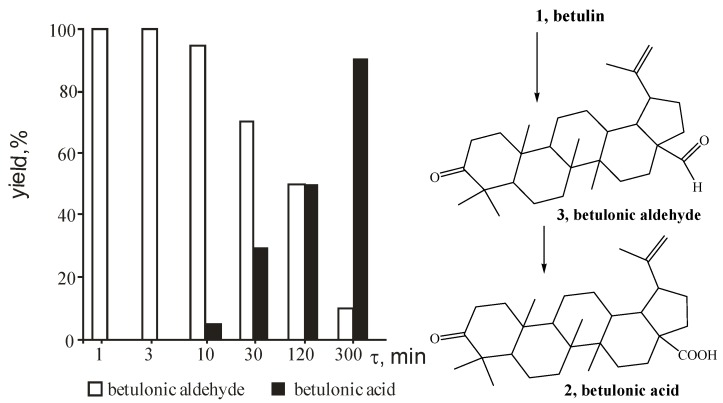
Diagram of yields of betulonic acid (black colour) and betulonic aldehyde (white colour) in time.

Surprisingly, oxidation of betulin (**1**) on silica gel for 30 min gave betulonic aldehyde (**3**) as a single product (Table 2, example 7) with 100% conversion of starting compound. Later on betulonic acid was obtained in the reaction mixture and at 8 hours the molecular ratio of betulonic aldehyde (**3**) to betulonic acid (**2**) became 1:1. The betulonic acid formation takes place as betulonic aldehyde oxidation. Assuming that a two-stage mechanism would be realized on alumina support we investigated betulin oxidation under the same conditions but at 0 °C. A two-step mechanism of betulin oxidation on Al_2_O_3_ was suggested, but the rate of betulonic acid formation was increased many times (Figure 2). 

**Figure 3:**
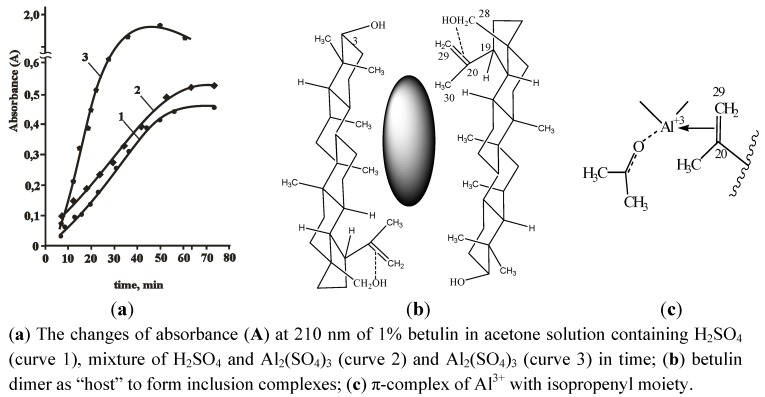
Betulin dissolving by chelation of components in the reaction medium.

The data of Table 2 may be explained by the influence of homogeneity in the reaction zone on the selectivity of betulin oxidation in the presence Al^3+^-ions on solid surface or in the solution. It was been shown that water solution of Al_2_(SO_4_)_3_ as well as H_2_SO_4__conc._ dissolve betulin (**1**) in ultra-sound dispersed suspension in acetone giving a transparent colourless solution. The absorbance band in the 190–220 nm region of the UV spectra of aqueous acetone solutions of betulin in the presence of H_2_SO_4_ or Al_2_(SO_4_)_3_ mixture was increasing with time up to 70 min and after that it became a constant value (Figure 3a, curves 1,2). The influence of Al_2_(SO_4_)_3_ on betulin dissolution was much more pronounced (Figure 3a, curve 3). 

It is common knowledge that selectivity is increased when homogeneity is achieved in the reaction zone [23]. Variation of the dispersion state of the reaction zone on a solid support may be due to the sorption of some components by the solid support, formation of intermediate products and implication of betulin in complexes with Al^3+^ having better solubility than complexes only with H_2_SO_4_. 

Such dissolution is typical for inclusion complexes of betulin derivatives with solvents such as benzene, toluene, isopropanol and several metal ions [24], while clathrates are formed by betulin dimers as "head-to-tail" structures. The interaction of H_2_SO_4_ with steroid compounds due to their solution and such types of reactions are usually used for identification of steroid rings. 

**Figure 4:**
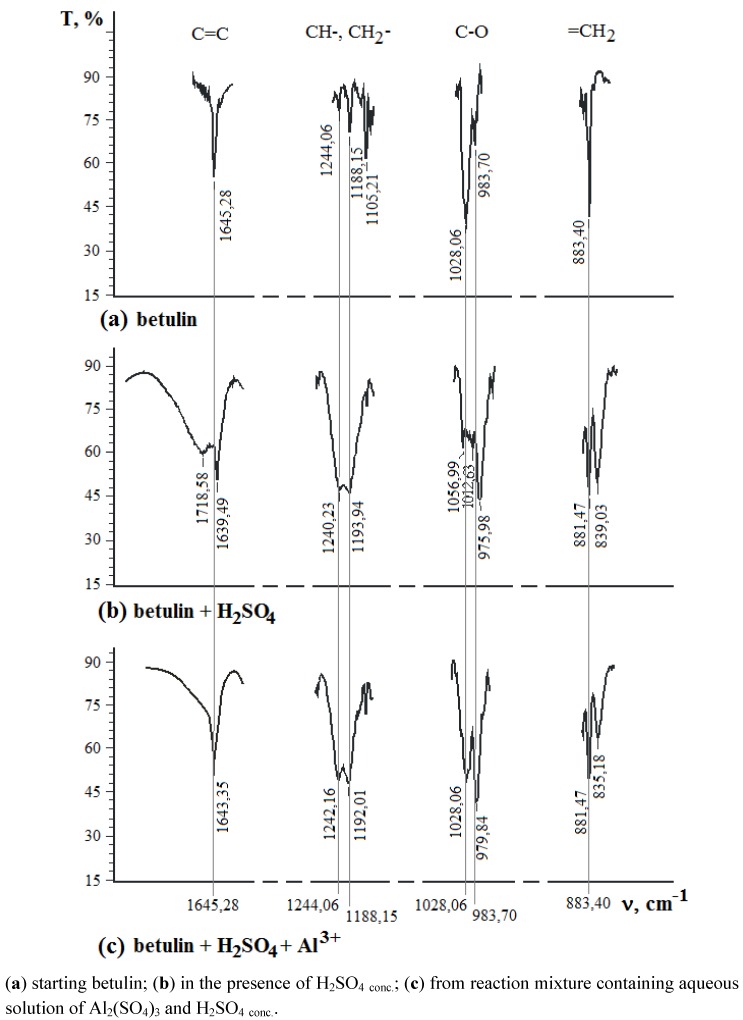
IR spectra of solid products evolved from 1% betulin solution in acetone.

It has been estimated that some red solid products were formed after betulin oxidation by conc. H_2_SO_4_ (3 mass %). The IR region (900–800 cm^−1^) relating to the double bond in the isopropenyl moiety of betulin was changed: one narrow band of 883 cm^−1^ was transformed into two bands at 881 cm^−1^ and 832 cm^−1^ and at the same time, the primary alcohol band (1028 cm^−1^) disappeared, but the bands of the epoxy group (1242 cm^−1^ and 976 cm^−1^) and of allobetulin (1056 cm^−1^) were established. Changes of the 1740–1640 cm^−1^ region (C=O, С=С) were noted too (Figure 4).

**Figure 5:**
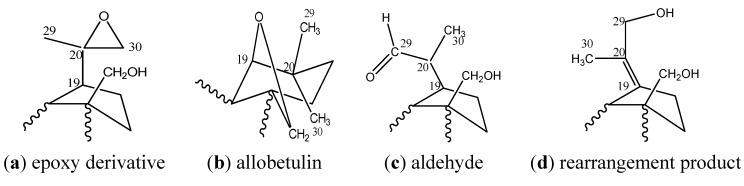
Proposed fragments of oxidation products of the isopropenyl moiety.

We suggest that changes to betulin by the action of H_2_SO_4_ occurred upon the oxidation of the isopropenyl moiety as well as the isomerization of the primary alcohol group. The most probable fragments of betulin derivatives are presented in Figure 5. This proposal has been corroborated with ^1^H-NMR data. The intensity of olefin protons signals in isopropenyl moiety in ^1^H-NMR spectra (δ = 4.71 ppm and δ = 4.59 ppm) was decreased two-fold. The intensity of signals of C-20 (δ = 150.24 ppm) and C-29 (δ = 109.46 ppm) in the ^13^C-NMR spectra was decreased too, but δ = 86.79 ppm, δ = 70.33 ppm and δ = 63.52 ppm signals were observed. 

In the presence of Al^3+^-ions (0.01%) the betulin oxidation up to allobetulin or other oxidation products was decreased (Figure 4). Aluminum as an element with a d-configuration with Lewis acid properties that may protect the isopropenyl moiety by formation of a π-complex (Figure 3c) in the presence of H_2_SO_4_ as well as Cr^6+^-H_2_SO_4_ system. 

The most important advantage of betulin oxidation when Al^3+^-ions were generated from Al_2_O_3_ was the ability to collect Cr3+ compounds as flakes by sorption or by other interactions on the solid surface. This result allows one to remove Cr^3+^-containing granules from reaction mixtures very easily, in contrast to the SiO_2_ support which can’t absorb Cr^3+^. 

Aluminium oxide surface layers in aqueous acetone medium are transformed under: (**1**) surface dissociation, (**2**) deprotonation and (**3**) dimerization via hydroxy-bridge joint into different kinds of ions and multinuclear cations respectively [25]: 


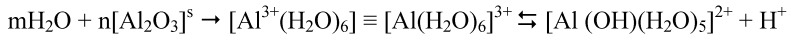



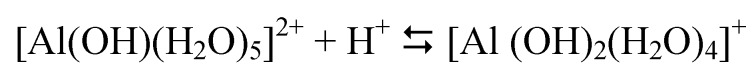



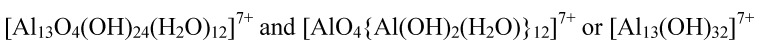


If such large amounts of sulfuric acid as in our experiments were used, it is most probable that reaction product type (2)—[Al(OH)_2_(H_2_O)_4_]+—ocurrs. The features of high reactivity of wet aluminium oxide surface with [Al(OH)_2_]^+^ or [Al(OH)_2_(H_2_O)_4_]+ in aqueous acetone medium containing K_2_Cr_2_O_7_–H_2_SO_4_ are due to: (i) anionic species of chromium derivatives with betulin; (ii) formation of inclusion complexes of reduced Cr^3+^-ions; (iii) solid solution due to replacement of Cr^3+^–Al_2_O_3_ [25,26].

**Scheme 1:**
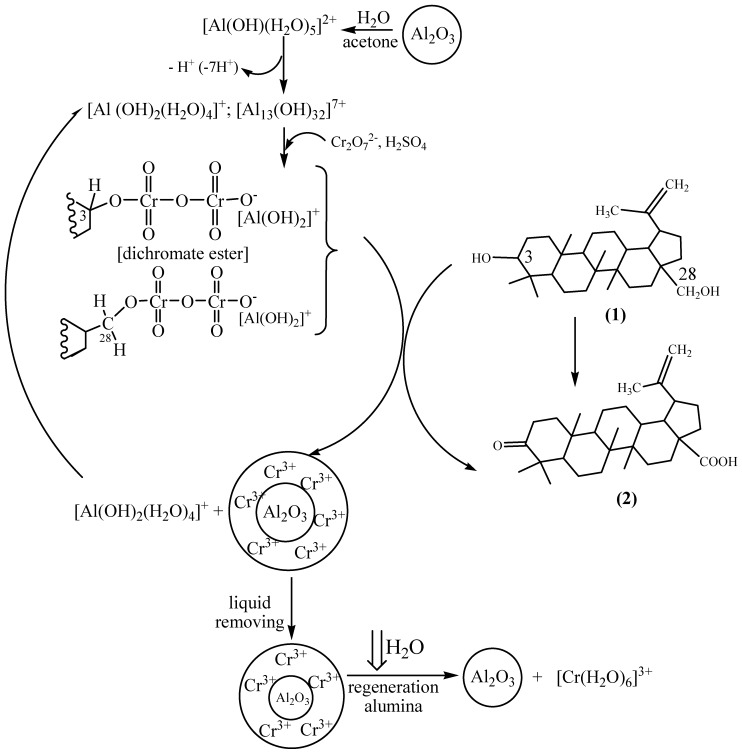
General formulation of synthesis of betulonic acid using oxidation of betulin on alumina in aqueous acetone by K_2_Cr_2_O_7_–H_2_SO_4_.

The improved homogeneity in the reaction zone may be explained by stabilization of chromium esters with betulin, formed according to a Westheimer mechanism [27] which is due to aluminium cations (Scheme 1).

The oxidation of betulin by K_2_Cr_2_O_7_–H_2_SO_4_ in aqueous acetone in the presence of Al^3+^ was studied in more a detail by UV spectrophotometry. A UV absorption characteristic of Cr^6+^ in different media occurs at λ_max_ = 350–360 nm. The absorbance (A) at a fixed wavelength follows the Beer-Lambert Law. Dichromate-ion may exist as the following forms: Cr_2_O_7_^2−^, HCrO_4_^−^, HCr_2_O_7_^−^. Lately HCr_2_O_7_^−^ has been considered as the main particle in K_2_Cr_2_O_7_–H_2_SO_4_ system [25,26].

**Figure 6:**
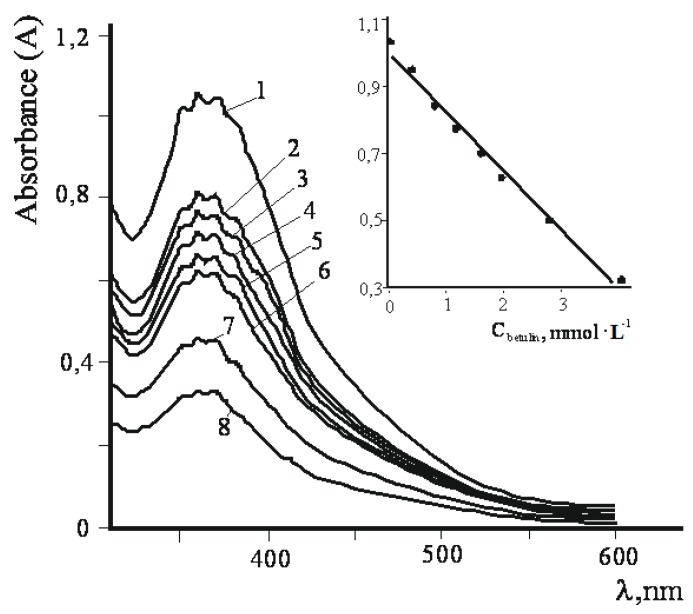
UV-visible spectra of reaction mixtures; C_K__2Cr__2O__7_ = const = 6.05 mmol·L^−1^; C_Al__2(SO__4)__3_ = const = 0.27 mmol·L^−1^. Initial concentration of betulin corresponds to N curves (mmol·L^−1^) 1–0; 2–0.40; 3–0.80; 4–1.21; 5–1.61; 6–2.01; 7–2.82; 8–4.03. The inset figure is magnification for the absorbance A: A = f (c_betulin_).

The reaction mixtures were at the constant concentration of oxidant (K_2_Cr_2_O_7_–H_2_SO_4_) and Al_2_(SO_4_)_3_ but with variable concentration of betulin. From Figure 6, the peak intensity at 360 nm decreased linearly as the concentration of betulin increased.

The decrease of the absorption at λ_max_ = 360 nm means the concentration of Cr^6+^ is decreasing and may be explained by the interaction of betulin (**1**) with Cr^6+^ – H_2_SO_4_ – oxidant resulting in formation chromate ester according to the Westheimer mechanism for the oxidation of alcohols [27]. The Westheimer mechanism involves the rapid initial formation of a chromate ester followed by the low rate determining, decomposition of the ester by removal of the α-proton by base B. In aqueous acetone the base was considered to be water. Some reports consider chromium oxidation in acid medium as a process of conversion to the protonated bimetallic chromium (VI) species, resultant in the formation of the monochromate ester which, under the decomposition in the rate-determining step, to give the product [25,27]. Monitoring the absorption at 360 nm in time A = f (τ) in the initial reaction mixtures was studied. The sampling was carried out from supernatant liquid using reaction mixture during synthesis of betulonic acid (**2**). Dilution of supernatant liquid by CH_3_CN – DMSO in a volume ratio of 10:1 conserved the homogeneity of the analyzed sample. 

**Figure 7:**
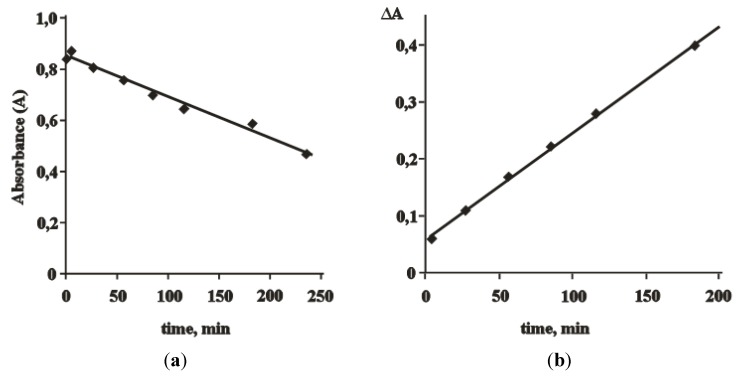
Dynamic range of the absorption at 360 nm (**a**) A = f (τ) during synthesis for supernatant liquid of reaction mixture under condition: C_betulin_ = 2.01 mmol·L^−1^; ν_Cr__6+_/ν_betulin_ = 3; m_betulin_/m_Al__2(SO__4)__3_ = 10. 20-times dilution of sample was carried out by CH_3_CN–DMSO in volume ratio as 10:1. (**b**) ΔA = f (τ), where ΔA = A_in_ − A_car_, A_in_ – initial absorption; A_car_ – the absorption in a time of synthesis.

The absorbance (**A**) at 360 nm was decreased 2-fold during 1.5–2 h (Figure 7), while 100% conversion of betulin (**1**) was achieved during this time. The analysis of the crude from supernatant liquid corresponded only to the betulonic acid (**2**). This result was due to the participation of only monochromate ester in betulin oxidation although dichromate ester was formed initially.

The value of ΔA depends proportionally on the concentration of Cr^6+^-containing complexes or ester with betulin. The linearity of absorbance against time plots means that the reaction was found to be of zero order with respect to Cr^6+^-containing compound mathematically and, most probably, had catalytic character. Actually, it is very difficult to estimate kinetic parameters in such multistage reactions passing through parallel and sequential reactions (ester or complex formation, oxidation).

## 3. Experimental

### 3.1. General

IR spectra were run from on a Shimadzu IR-Prestige-21 instrument (KBr tablets). The ^1^H- and ^13^C-NMR spectra were recorded on a Bruker Advance DPX–200 or a Bruker DRX SF-500 spectrometers in DMSO-*d_6_* solution using TMS as an internal standard. The UV-Vis spectra were obtained on a Analytik Jena Specord S-100 instrument. Melting points were measured using the capillary tube method on an Electrothermal 9200 apparatus. HPLC analysis were done using a Shimadzu LC-10 Avp equipped with a Discovery C_18_ column (250 × 4.6 mm, 5μm) and UV-detector. The eluent was acetonitrile—water 80:20 (υ/υ) as isocratic mobile phase at a flow rate of 1 mL·min^−1^ at 25 ^ο^C, the injection volume was 20 μL, the detection was accomplished at 210 nm, and the analysis time was 15 min. Residual traces of Al (III) and Cr (VI) were determined by a Shimadzu AA 7000 Atomic Absorption spectrophotometer using standard solutions. Electron-impact mass spectra (EI-MS) were obtained on a JMS-HX 110 mass spectrometer.

### 3.2. Materials and Reagents

Deionized water (pH 5.6, χ 18mSm) was used, solvents (acetone, isopropanol, methanol) were purified by known methods. As a solid support we used silica gel 60 (15–49, 40–63 and 63–200 μm, Merck), Alumina - γ-Al_2_O_3_ (Aldrich), zeolite 13X, NaA (270, 325 and 400 mesh, Merck). “Wet” solid support was formed by impregnation of oxidant (Cr^6+^-compound–H_2_SO_4_).

Betulin (**1**,C_30_H_50_O_2_) was prepared according to the literature method [28], m.p. 260 °C (lit. 254–256 °C [28]); purity 99.5%, IR, ν, cm^−1^: 3470 st (OH), 1640 st (C=C); ^1^H-NMR δ, ppm: 4.67 m (1H, =CH_2_), 4.57 m (1H, =CH_2_), 3.78 br. s (1H, 28-CH_2_OH), 3.31 m (1H, 28-CH_2_OH), 3.17 m (1H, 3-CHOH), 2.36 m (1H, 19-CH), 1.66 s (3H, CH_3_), 1.23 s (3H, CH_3_), 0.96 s (3H, CH_3_), 0.94 s (3H, CH_3_), 0.80 s (3H, CH_3_), 0.74 s (3H, CH_3_). ^13^C-NMR, δ, ppm: 76.71 (C-3), 109.46 (C-29), 150.24 (C-20), 57.87 (C-28). EI-MS *m/z* (%): 442 (M^+^, 40), 411(60), 203 (95), 189 (100), 95 (85).

### 3.3. Oxidation of Betulin (*1*) to Betulonic Acid (*2*) by K_2_Cr_2_O_7_–H_2_SO_4_ on Alumina

Granules of Al_2_O_3_ (9.2 g, A_sp_ = 500 m^2^/g) was treated with potassium dichromate solution (3 g, 10 mmol), H_2_O (20 mL) and conc. H_2_SO_4_ (5 mL) in sequence. Betulin (**1**, 1.5 g, 3.4 mmol) was dispersed by ultra-sound in acetone (138 mL) to give a white suspension. Oxidant with “wet” alumina was added to the suspension of betulin in a reaction flask, then the reaction mixture was stirred for 1.5 h (HPLC-control). After the residue of inorganic compounds was removed, water (500 mL) was added to the liquid phase. Crude betulonic acid (**2**) precipitated (as white flakes) and after filtration **2** was washed multiple times with hot water. The precipitate (1.48 g, 97% yield) was recrystallized from methanol. The crystals were isolated and dried in a vacuum oven to afford pure (>95% by HPLC) **2**, m.p. 250–52 °C (methanol) (lit. 245–248 °C [6]). IR, ν, cm^−1^: 1705 st (C=O), 1641 st (С=С); 883 st (=CH_2_); ^1^Н-NMR δ, ppm: 4.68 s (1H, 29-H), 4.55 s (1H, 29-H), 2.23 m (1H, 19-H), 1.65 s (3H, 30-CH_3_), 1.02–1.95 (3H, complex, CH_2_, CH), 1.02 s (3H, 26-CH_3_), 1.00 s (3H, 25-CH_3_), 0.98 s (3H, 27-CH_3_), 0.86 s (3H, 23-CH_3_), 0.85 s (3H, 24-CH_3_). ^13^C-NMR δ, ppm: 216.52 (C-3), 109.67 (C-29), 150.33 (C-20), 177.26 (C-28). EI-MS *m/z* (%): 454 (M^+^, 58), 248 (64), 219 (42), 205(76), 189 (88), 136 (100), 121 (90). The precipitate of betulonic acid after recrystallisation (methanol) didn’t include any residual traces of Cr and Al.

### 3.4. Oxidation of Betulin by K_2_Cr_2_O_7_–H_2_SO_4_ on Silica Gel

Granules of SiO_2_ (10 g, A_sp_ = 800 m^2^/g) were treated with potassium dichromate solution (1.5 g, 5 mmol), H_2_O (40 mL) and conc. H_2_SO_4_ (2.5 mL) in sequence. Betulin (**1**, 1 g, 2.3 mmol) was dispersed by ultra-sound in acetone (40 mL) to give a white suspension. Oxidant with “wet” SiO_2_ was added to suspension of betulin in a reaction flask, then the reaction mixture was stirred during 10 min (HPLC-control). After that the SiO_2_ was removed and water (500 mL) was added to the liquid phase. The white flake precipitate was filtered, washed with hot water and was dried. The finished product was identified as betulonic aldehyde (**3**), m.p. 163–165 °C (lit. 165–166 °C [29]). IR, ν, cm^−1^: 1730–1728 st (C=O), 1641 st (С=С); 883 st (=CH_2_); ^1^Н-NMR δ, ppm: 9.67 s (1Н, 28-СНО), 4.68 s (1H, 29-H), 4.55 s (1H, 29-H), 2.99 dd (1H, 3α-H), 2.23 m (1H, 19-H), 1.65 s (3H, 30-CH_3_), 1.02–1.95 (3H, complex, CH_2_, CH), 1.02 s (3H, 26-CH_3_), 1.00 s (3H, 25-CH_3_), 0.98 s (3H, 27-CH_3_), 0.86 s (3H, с, 23-CH_3_), 0.85 s (3H, с, 24-CH_3_); ^13^C-NMR δ, ppm: 216.52 (C-3), 109.67 (C-29), 150.33 (C-20), 206.55 (C-28). EI-MS *m/z* (%): 438 (11.5), [M-CHO]^+^ 409 (20.0), 273 (3.2), 219 (20.2), 205 (38.4), 189 (39.6), 133 (35.6), 105 (55.8), 81 (65), 55 (100).

### 3.5. Synthesis of Betulinic acid Using Crude Betulonic Acid (*2*)

The crude betulonic acid (**2**) was subjected to NaBH_4_ (THF or isopropanol) reduction (10 molar ratio to betulonic acid) at room temperature to yield a mixture (5:95) of 3α- and 3β-betulinic acid (1.4 g), m.p. 295–300 ^ο^C (methanol), after recrystallization m.p. 310–311 °C (lit. 290–293 °C [30]). IR, ν, cm^−1^: 3450 st (OH), 1683 st (C=O), 1641 st (С=С); 883 st (=CH_2_); 1031, 1042 st (α, β-3-C–OH). ^1^Н-NMR δ, ppm: 4.68 s (1H, 29-H), 4.55 s (1H, 29-H), 3.24 t (1 H, 3β-H), 2.99 dd (1H, 3α-H), 2.23 m (1H, 19-H), 1.65 s (3H, 30-CH_3_), 1.02–1.95 (3H, complex, CH_2_, CH), 1.02 s (3H, 26-CH_3_), 1.00 s (3H, 25-CH_3_), 0.98 s (3H, 27-CH_3_), 0.86 s (3H, 23-CH_3_), 0.85 s (3H, 24-CH_3_). ^13^C-NMR δ, ppm: 76.83 (C-3), 109.67 (C-29), 150.33 (C-20), 177.26 (C-28). EI-MS *m/z* (%): 457 (M^+^+H, 7), 307 (25), 154 (100), 136 (60). The precipitate of betulonic acid after recrystallization (methanol) didn’t include anyresidual traces of Cr and Al.

## 4. Conclusions

We have developed the effective methods for selective oxidation of betulin up to carbonyl derivatives. K_2_Cr_2_O_7_–H_2_SO_4_ oxidizes betulin to betulonic acid in aqueous acetone at 15–25 °C with 93%–98% yields in the presence of Al^3+^-ions. The same procedure using silica gel gives a single product—betulonic aldehyde—after 30 min.

The high selectivity of oxidation is determined by the protection of the interaction of betulin with Si^4+^ on silica gel surface or by π-complex formation of Al^3+^-ions as Lewis acid with terminal the double bond of the isopropenyl moiety. An alternative reason for the high selectivity may be complexes formed by betulin with chromium intermediate products, for example chromium ester, stabilized by [Al(OH)_2_]^+^-ions. The main advantage of this procedure is the possibility to remove toxic Cr^3+^ from reaction mixtures by sorption on alumina and provide a simple and environmentally friendly reaction, resulting in a less a hazardous method for betulin oxidation.
